# Clinical exome sequencing reveals a mutation in *PDHA1* in Leigh syndrome: A case of a Chinese boy with lethal neuropathy

**DOI:** 10.1002/mgg3.1651

**Published:** 2021-03-04

**Authors:** Ke Gong, Li Xie, Zhong‐shi Wu, Xia Xie, Xing‐xing Zhang, Jin‐Lan Chen

**Affiliations:** ^1^ Department of Cardiovascular Surgery The Second Xiangya Hospital of Central South University Central South University Changsha China; ^2^ Department of Pediatrics The Second Xiangya Hospital of Central South University Central South University Changsha China

**Keywords:** clinical exome sequencing, Guillain–Barré syndrome, Leigh syndrome, neurodegenerative disease, *PDHA1* mutation

## Abstract

**Background:**

Leigh syndrome, the most common mitochondrial syndrome in pediatrics, has diverse clinical manifestations and is genetically heterogeneous. Pathogenic mutations in more than 75 genes of two genomes (mitochondrial and nuclear) have been identified. *PDHA1* encoding the E1 alpha subunit is an X‐chromosome gene whose mutations cause pyruvate dehydrogenase complex deficiency.

**Methods:**

Here, we have described a 12‐year‐old boy with lethal neuropathy who almost died of a sudden loss of breathing and successive cardiac arrest. Extracorporeal membrane oxygenation rescued his life. His diagnosis was corrected from Guillain–Barré syndrome to Leigh syndrome 1 month later by clinical exome sequencing. Furthermore, we used software to predict the protein structure caused by frameshift mutations. We treated the boy with vitamin B1, coenzyme Q10, and a ketogenic diet.

**Results:**

A *PDHA1* mutation (NM_000284.4:c.1167_1170del) was identified as the underlying cause. The amino acid mutation was p.Ser390LysfsTer33. Moreover, the protein structure prediction results suggested that the protein structure has changed. The parents of the child were negative, so the mutation was de novo. The comprehensive assessment of the mutation was pathogenic. His condition gradually improved after receiving treatment.

**Conclusion:**

This case suggests that gene detection should be popularized to improve diagnosis accuracy, especially in developing countries such as China.

## Graphical abstract

Here, we describe a 12‐year‐old boy with a lethal neuropathy who almost died of a sudden loss of breathing and successive cardiac arrest. One month later, we found that the child had a de novo mutation (NM_000284.4: c.1167_1170del) in the PDHA1 gene through clinical exome sequencing, and corrected his diagnosis from Guillain‐Barré syndrome to Leigh syndrome. This case shows that genetic detection should be popularized to improve diagnostic accuracy, especially in developing countries such as China.

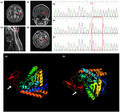

## 1 INTRODUCTION

Leigh syndrome (MIM 256,000; LS) is a progressive neurodegenerative disease caused by defects in mitochondrial oxidative phosphorylation and is the most common mitochondrial syndrome in pediatrics (Baertling et al., 2014). The incidence of LS is estimated to be 1:77,000–1:34,000 live births (Piekutowska‐Abramczuk, 2008). Currently, no precise data have been published on the incidence of LS in China (Zhang, Yang, et al., 2007). Since the identification of the first pathogenic mutation in a patient with LS in 1991, pathogenic mutations in more than 75 genes of two genomes (mitochondrial and nuclear) have been identified (Lake et al., 2016). *PDHA1* (OMIM 300502) is an X‐chromosome gene, and *PDHA1* mutation is the main reason for Pyruvate dehydrogenase complex deficiency (PDCD), and about 25% of PDCD can cause LS (DeBrosse et al., 2012; Patel et al., 2012).

LS is clinically heterogeneous with significant differences in age of onset, age of death, and symptoms. A preliminary clinical diagnosis can be based on clinical manifestations, physical and biochemical examinations, and head magnetic resonance imaging (MRI) results. Furthermore, genetic testing can provide an auxiliary diagnosis. Although treatment with vitamin B1, coenzyme Q10, and a ketogenic diet (KD) will help, the treatment effect is usually unsatisfactory due to LS's poor prognosis (Chang et al., 2020; Ogawa et al., 2020; Sofou et al., 2014).

## 2 CASE REPORT

### 2.1 Editorial policies and ethical considerations

#### 2.1.1 Ethics approval and consent to participate

Not applicable.

#### 2.1.2 Patient consent for publication

The patient information in this case report has been approved by the patient guardian, and he understands the content of the article.

### 2.2 Clinical report

A 12‐year‐old boy was admitted to a local hospital because of fever and weakness. However, his condition deteriorated rapidly, and he was transferred to the pediatric intensive care unit when he developed difficulty breathing. He stopped breathing 2 h later, and an artificial airway with mechanical ventilation was established. However, another 2 h later, his heart stopped suddenly. Cardiopulmonary resuscitation (CPR) was not successful. The extracorporeal membrane oxygenation (ECMO) team at our hospital was called, and he was transferred to our hospital under ECMO support for further treatment.

The child had been healthy until he was 2 years old. His birth history was normal. His parents were not consanguineous, and they were healthy. When he was 2.5 years old, he walked with a staggering gait and fell often. As a result, the child was immediately checked and treated at the local hospital. The doctors first performed routine biochemical and physical examinations, and the examination revealed that the child's knee reflex disappeared. Electromyography (ECG) suggests peripheral neuropathy, and it is mainly sensory nerve damage. At the same time, the child had symptoms of repeated muscle weakness. Therefore, the child was diagnosed with Guillain–Barré syndrome (GBS) (Hughes & Cornblath, 2005). High‐dose immunoglobulin was administered for 3 days, and his condition improved. Mecobalamin, citicoline, and adenosine triphosphate were administered over a long period to restore his cranial nerves. However, he was hospitalized due to muscle weakness again when he was 6 years old and again diagnosed with GBS.

**FIGURE 1:**
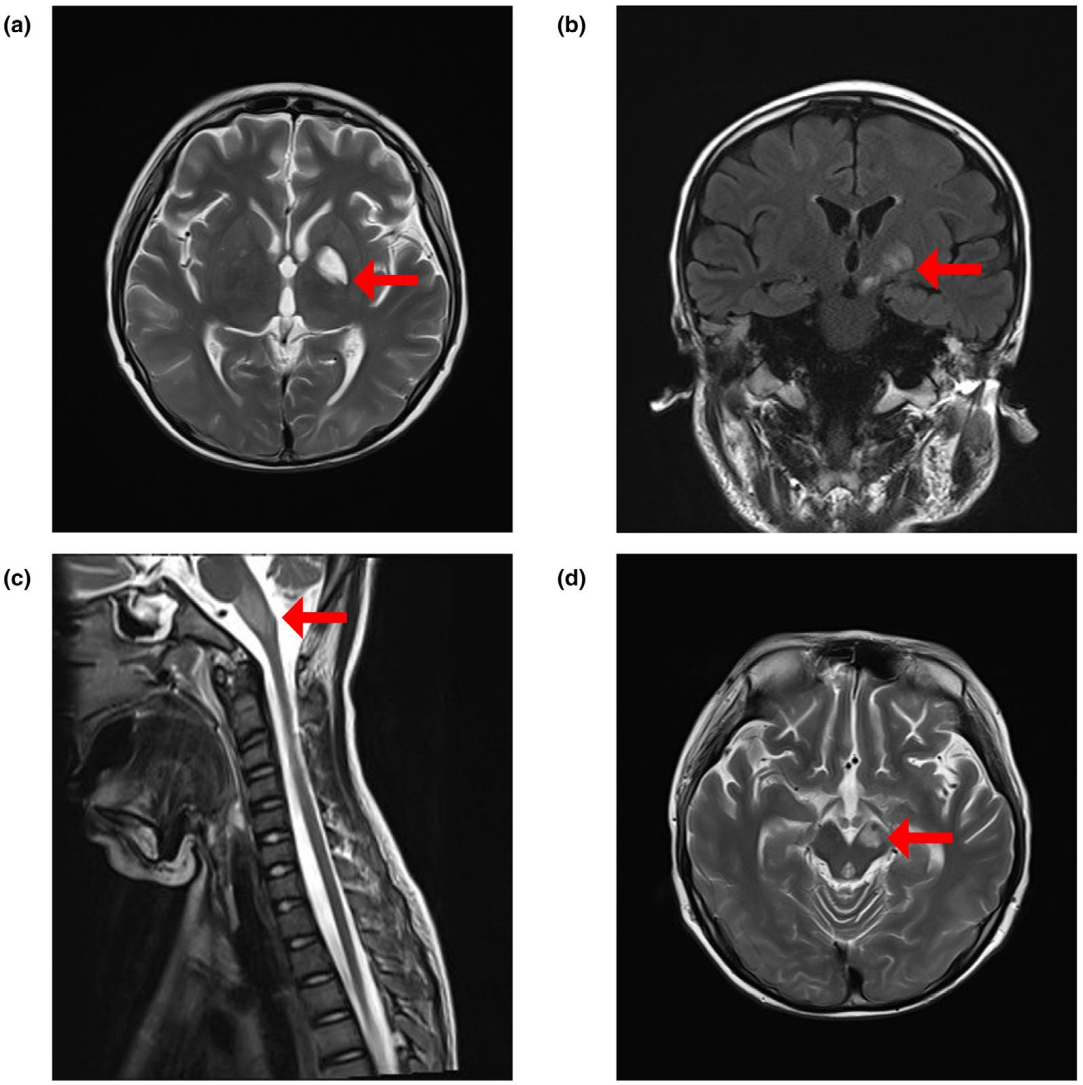
MRI of the head and spinal cord showing multiple lesions. (a and b) Axial T2‐weighted and coronal FLAIR brain MRI shows hyperintensity in the left basal ganglia region (red arrow). (c) Sagittal T2‐weighted cervical spine MRI shows hyperintensity in the medulla oblongata (red arrow). (d) Axial T2‐weighted brain MRI shows hyperintensity in the left midbrain (red arrow)

Three months before arriving at our hospital, the boy almost died of sudden cardiac arrest. The ECMO team rescued and stabilized his circulation and breathing. Brainstem lesions were suggested on brain computed tomography and MRI (Figure 1). EEG examination suggested that background activity slowed down. The child had a lumbar puncture. A cerebrospinal fluid (CSF) examination showed normal protein levels (290.17 mg/L; N: 150–450 mg/L) with leukocytosis (WBC 14 × 10^6^/L). And the child had symptoms of ataxia and weakened tendon reflexes. Although there was no protein‐cell separation phenomenon, a pediatrician and a neurological physician highly suspected that he had the most severe GBS‐MFS (Miller–Fisher syndrome). The boy and his parents underwent clinical exome sequencing (CES) to exclude genetic metabolic diseases.

As GBS could not be excluded, immunoglobulin was injected for 5 days, with a total dose of 20 g, but the boy's clinical symptoms did not improve significantly. ECMO was continued for 4 days until his heart function and breathing recovered completely. He regained his consciousness gradually, but his myodynamia was so weak that he could not move his limbs, and the ventilator could not be weaned off because of weak spontaneous breathing.

During hospitalization, laboratory tests and examinations were conducted and yielded the following results: Blood amino acid and ester acylcarnitine spectrum were normal. During fasting, plasma lactate levels were 9.8 mmol/L (N: 0.5–1.7 mmol/L). ECG revealed peripheral neurogenic damage; multiple demyelinating mixed axonal damage to the proximal nerve roots and distal nerve fibers and damage to the motor and sensory nerve fibers of both limbs; the lower limbs were worse than the upper limbs. A clear spontaneous potential was observed, which suggested that there was actual damage. Head MRI revealed abnormal signal intensity in the left basal ganglia, left midbrain, fourth ventricle, cerebellar dentate nucleus, and cervical spinal cord (Figure 1).

Although the examination indicated a neurological disease, the targeted treatment did not improve the boy's clinical symptoms during the 1‐month hospitalization until the diagnosis was corrected through CES. CES suggests that the child has pyruvate dehydrogenase‐E1 alpha deficiency, resulting in LS. Thus, the KD was started, and medications such as coenzyme Q10, vitamin B1, and levocarnitine were administered. After the second 1‐month treatment, the boy's muscle strength was significantly improved, and he could lift his upper limbs. However, the muscle strength in his legs was still weak.

### 2.3 CES (5000‐gene panel)

**FIGURE 2:**
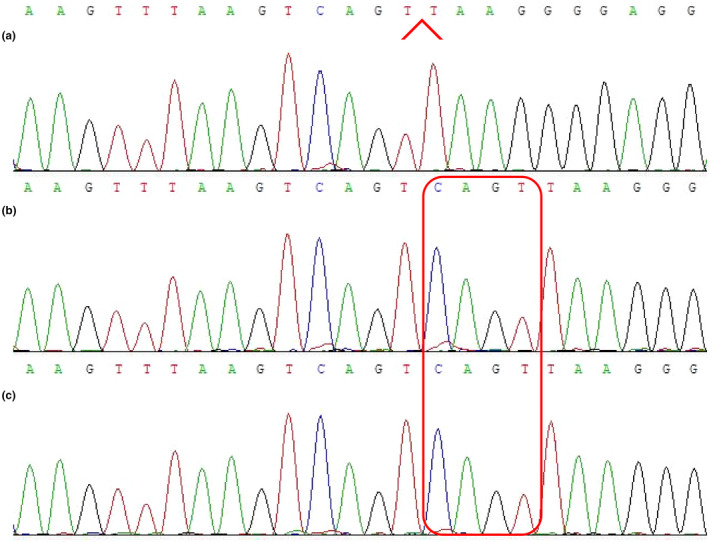
Sanger sequencing results. (a) Sequencing reveals a hemizygous mutation (NM_000284.4:c.1167_1170del mutation) in the patient. The mutation was identified at the red arrow in the *PDHA1* and resulted in the deletion of basic CAGT, at the chromosomal location chrX:19377758. (b and c) Sequencing results of the patient's father and mother

CES involves an application of next‐generation sequencing (NGS) that focuses on genes that have been found to be associated with diseases and reported in the Human Mutation Database® (Su et al., 2011). This subset of the exome currently contains approximately 5000 genes (25% of the exome) and is continuously expanding. The main advantage of CES is that it reduces data analysis, interpretation, and turnaround times and increases cost‐effectiveness by focusing on clinically characterized genes, applying trio analysis and improving data quality. The CES results for the boy's parents were normal, but a mutation in *PDHA1* was found in the patient. The mutation NM_000284.4:c.1167_1170del was found to be located in the 11th exon of *PDHA1*, which was located at chrX:19377758 (genome version: hg19) transcript NM_000284.4 (Figure 2). The location of this deletion mutation is a repeating sequence, so we named the deletion according to the HGVS naming rules. So the mutation resulted in NM_000284.4:c.1167_1170del and the deletion of the base CAGT. Sanger sequencing was performed and confirmed the deletion. This mutation is consistent with the genetic disease model, and the associated disease, a syndrome caused by E1 alpha pyruvate dehydrogenase deficiency, according to the ClinVar database, is consistent with the boy's clinical manifestations.

The following is the process of the pathogenicity of the variant of the disease according to the ACMG standard (Abou Tayoun et al., 2018; Richards et al., 2015). The disease and pathogenicity rating corresponding to this locus in the ClinVar database was E1‐α pyruvate dehydrogenase deficiency (pathogenic). PVS1 was chosen because this gene was a gene whose loss of function (LoF) was a known mechanism of disease. Since the mutated gene in this child was a gene with a known disease mechanism, PSV1 was chosen. However, because the mutation site was located at the 3'end and there were multiple transcripts, this evidence selection needed to be considered carefully. The first was that the gene had haploinsufficiency. The ClinGen haploinsufficiency score was 3, and ExAC pLI score was 0.992, which indicated that there was sufficient evidence for the PDHA1 gene to indicate haploinsufficiency (https://dosage.clinicalgenome.org/clingen_gene.cgi?sym=pdha1&subject). And the selected transcript was a biologically relevant transcript. The population frequency at this site was 0.00001 (the maximum value of ESP6500, 1000 g, and EXAC_ALL). The frequency of the Middle East Asian population in the gnomAD database was 0. The incidence rate in the normal population was very low. In addition, protein structure prediction and software prediction functions indicated changes in protein integrity. The above evidence supported the choice of PSV1. But it was not clear whether the altered region was critical to protein function. After comprehensive consideration, there was insufficient evidence for the direct selection of PVS1. Therefore, according to the strength level of ClinGen Sequence Variation Interpretation Working Group (ClinGen SVI) on PSV1, the strength of PSV1 needed to be determined in detail. In the next step, the exons affected by the mutation were neither enriched in high‐frequency LOF variants in the general population (ESP6500, 1000 g, and EXAC_ALL), and the exon was in biologically relevant transcripts. In the last step, the mutation removed less than 10% of the protein, so our PSV1 strength level was PVS1_Moderate. According to the instructions of the ClinGen SVI, the evidence level of PVS1 needed to be downgraded to PVS1_Moderate (Abou Tayoun et al., 2018). Because the mutation in the child was the same as the reported related mutation site and protein change, PS1 evidence should be selected. And the mutation was de novo, so PS2 evidence was chosen. In summary, refer to the ACMG gene mutation interpretation guide, the site met 3 pieces of evidence (PS1, PS2, and PVS1_Moderate), and the classification was assessed as Pathogenic (Richards et al., 2015).

### 2.4 Protein structure prediction

Comparison analysis of the primary structure of the normal protein and mutant protein: The mutationtasting website (http://www.mutationtaster.org/index.html) was used to predict the structure of gene mutations. The ExPASY software's Protparam tool (http://www.expasy.org/tools/protparam.html) was used to calculate the main physical and chemical properties. TMpred software (http://www. ch.embnet.org/software/TMPRED_form.html) was used to analyze the hydrophilicity and hydrophobicity of proteins and the analysis of protein transmembrane regions.

Comparison and analysis of the secondary structure of the normal protein and mutant protein: Predictprotein software (https://www.predictprotein.org/) was used to predict the secondary structure of the normal protein and mutant protein to determine whether there are structural changes.

Comparative analysis of the tertiary structure of the normal protein and mutant protein: Application of Phyre (http://www.sbg.bio.ic.ac.uk/~phyre/) predicts and compares the tertiary structure of the normal protein and mutant protein and roughly judge the impact of the mutation on the tertiary structure of the protein.

### 2.5 Protein structure prediction results

**FIGURE 3:**
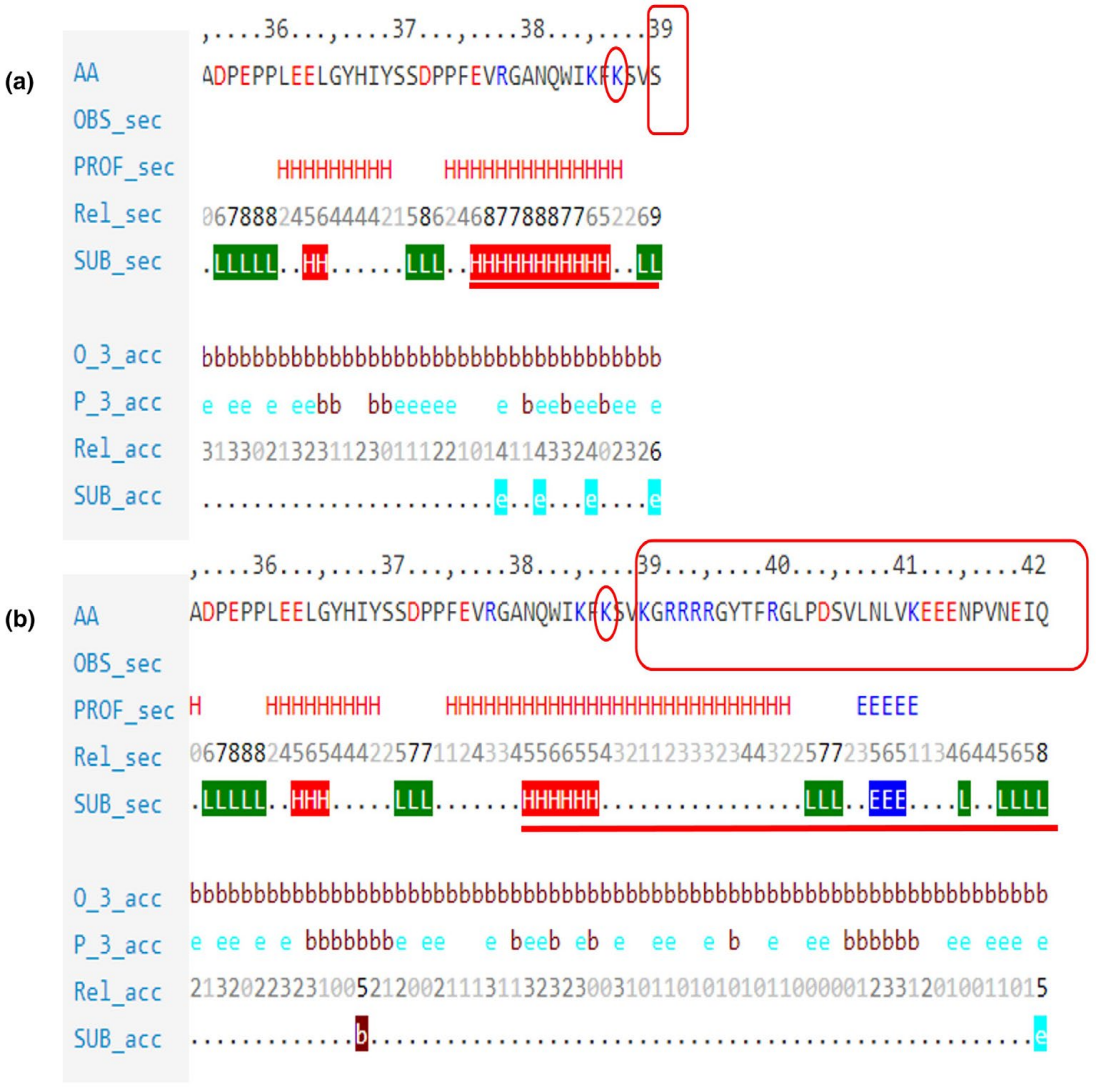
Comparison and analysis of the secondary structure of normal protein and mutant protein. (a) Wild type amino acid sequence. The Lys circled in red indicated that the frameshift mutation has changed from that position. (b) Mutant amino acid sequence. The red rectangles indicated the increased and changed amino acid sequence caused by splicing mutations compared with the normal ones. The part marked by the red line was the difference in secondary structure between the wild type and the mutant. The first three amino acids at the beginning of the gene frameshift mutation site were Lys, Ser and Val the same as the wild type. In fact, for the amino acid sequence, the first amino acid change was at the fourth amino acid. Lys replaced Ser, and the amino acid sequence behind it continued to extend until the next stop site. OBS_sec: Observations of secondary structure. PROF_sec: Predicted secondary structure. Rel_sec: The reliability of the predicted secondary structure. SUB_sec: A collection of predicted secondary structures. H: Helix, E: folding, L: Coiled. O_3_acc: Observations of relatively hydrophilic surfaces. P _3_acc: Predicted observations of relatively hydrophilic surfaces. Rel_acc: The reliability of the predicted hydrophilic surface. SUB_acc: A collection of predicted hydrophilic surfaces

**FIGURE 4:**
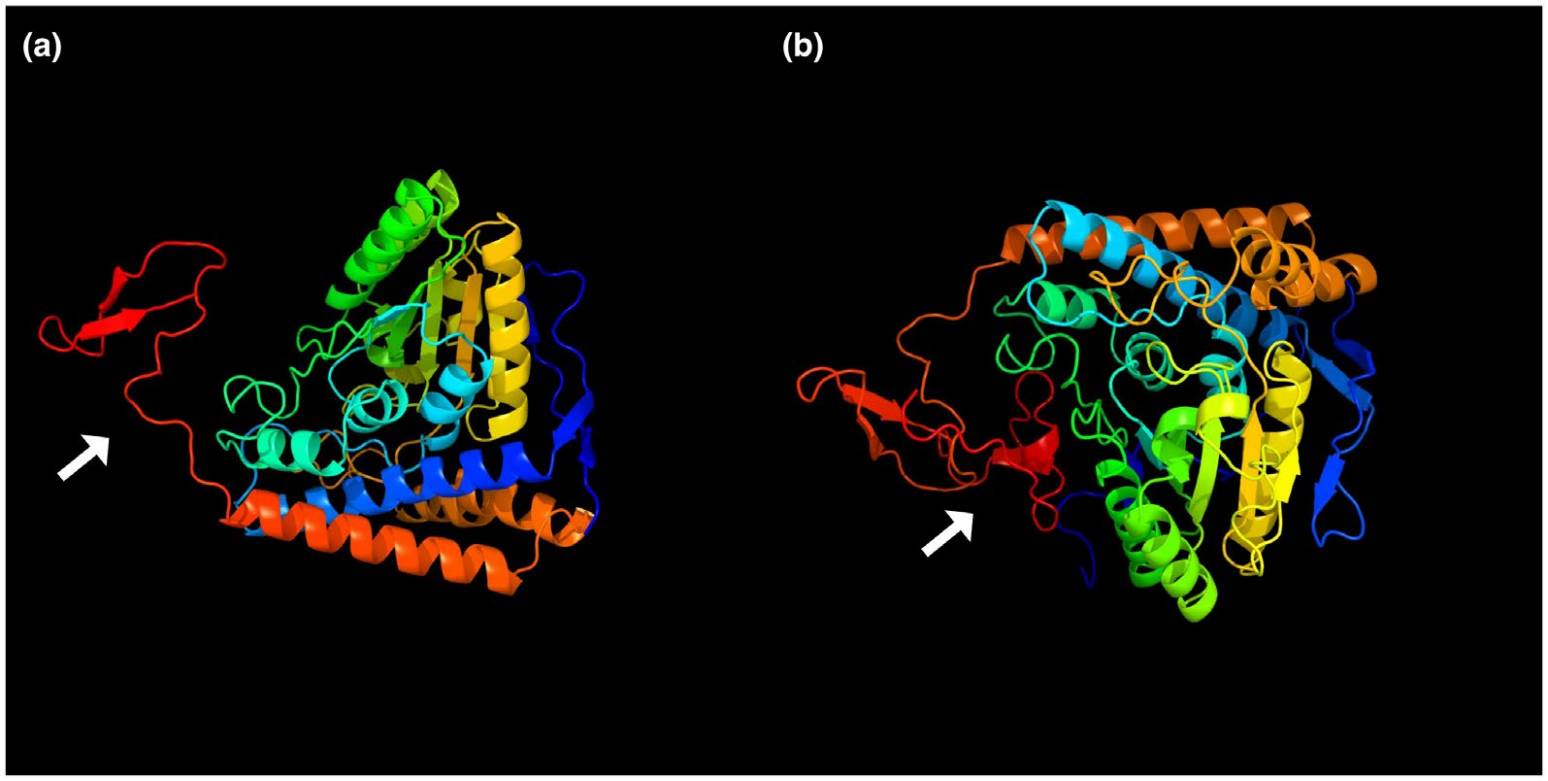
Comparative analysis of the tertiary structure of the normal protein and mutant protein. (a) Wild type, (b) Mutant type. Compared with the wild type, the mutant type has changed the structure of the protein end, as indicated by the white arrow

The prediction results showed that the mutation caused an amino acid frameshift variant (p.Ser390LysfsTer33). The results of comparative prediction and analysis of primary protein structure: E1 alpha subunit protein was composed of 390 amino acids. After the mutation, 31 amino acids were added to the amino acid sequence, the molecular structure was changed, the Molecular weight increased, the Extinction coefficient increased, and the Grand average of hydropathicity of the protein increased (Table 1). It was predicted that the *PDHA1* protein had two transmembrane regions, and this mutation was not in the transmembrane region. After the mutation, the transmembrane spiral structure of the protein remained unchanged. The comparison results of protein secondary and tertiary structure were shown in Figures 3 and 4. The results all suggested a structural change at the end of the protein.

**TABLE 1:** Predicted results of protein primary structure comparison

	Number of amino acids	Molecular weight	Formula	Total number of atoms	Theoretical pI	Extinction coefficients	Grand average of hydropathicity	Aliphatic index	Estimated half‐life	Instability index
Wildtype	390	43295.63	C_1899_H_3010_N_540_O_566_S_26_	6041	8.35	38570	−0.312	77.59	30 h	33.06
Mutant	421	46961.79	C_2059_H_3272_N_592_O_613_S_26_	6562	8.59	40060	−0.376	77.65	30 h	37.72

## 3 DISCUSSION

The British neuroscientist Denis Leigh first described LS in 1951 (Leigh, 1951). LS has a variety of clinical manifestations, ranging from asymptomatic to severe neurological problems. Usually, the onset is within 2 years after birth (Sofou et al., 2014) and occurs during an infection or disease after the early stages of normal development. Progression is usually rapid; most deaths occur before the age of 3, but some will get sick and die at birth, and some cases can survive for a long time until adulthood (Ma et al., 2013; Montpetit et al., 1971; Rahman et al., 1996; Van Erven et al., 1987). Adult‐onset LS has rarely been reported (McKelvie et al., 2012). Patients develop neurological symptoms, including retardations in mental growth and development, muscle weakness, and ataxia, and a small number of patients with nystagmus and optic atrophy have been reported. In addition to common symptoms, LS may be manifested in multiple system involvements (López et al., 2006; Monlleo‐Neila et al., 2013; Sofou et al., 2014). The characteristic manifestation of LS is that the decline of nervous system functions is related to bilateral symmetrical lesions in the brain stem and basal ganglia (Lake et al., 2015; Montpetit et al., 1971). On T2‐weighted head MRI, lesions in these areas show a high focal signal. Moreover, lactic acid elevations can be detected on magnetic resonance spectroscopy. In addition, there are some rare abnormalities, including white matter involvement and brain atrophy (Arii & Tanabe, 2000). Patients with LS sometimes show occasional peripheral muscle weakness that mimics GBS at an early stage (Strassburg et al., 2006). The typical manifestations of GBS are features of peripheral radiculitis and protein‐cell separation. In this case, despite the lack of protein cell separation, the boy was diagnosed with GBS early due to his progressive muscle weakness and nerve damage. However, the child's symptoms did not improve after treatment, according to GBS treatment. At the same time, genetic testing suggested PDCD. Combined with clinical symptoms, we finally diagnosed it as LS.

LS shows extensive genetic heterogeneity. More than 75 genes in nuclear and mitochondrial genomes have been found to be associated with LS; these genes are involved in energy metabolism, such as in the mitochondrial respiratory chain complexes I, II, III, IV, and V. They are also involved in oxidative phosphorylation (OXPHOS) and the generation of ATP (Lake et al., 2016). In addition, *PDHA1* is an important part of the nuclear genome, and mutations in *PDHA1* can cause PDCD. Approximately 25% of patients with PDCD have LS (DeBrosse et al., 2012; Patel et al., 2012).

Pyruvate dehydrogenase complex (PDHc) contains three enzyme components (pyruvate dehydrogenase, dihydrogenamide acetyltransferase, and lipoamide dehydrogenase), two regulatory proteins (E1 kinase and E1 phosphatase), and the E3 binding protein. The PDH activity of the vast majority of patients is reduced, and the PDHc activity of some patients with PDCD is even <10% (Finsterer, 2008). A decrease in PDHc activity results in a decrease in the amount of pyruvate that is converted to acetyl‐CoA, which inhibits the tricarboxylic acid (TCA) cycle activity. As a result, the levels of NADH and FADH2 in cells are reduced, both of which are critical for the proper functioning of the mitochondrial respiratory chain. To date, mutations in patients with LS have been described in two structural subunits of the pyruvate dehydrogenase component (*PDHA1* and *PDHB*), in the gene encoding the lipoamide dehydrogenase (*DLD*), and in the E3 binding protein (*PDHX*). *PDHA1* mutations cause about 77.2% of all genetically diagnosed cases of PDCD, and the gene has identified more than 190 different mutations through the Human Gene Mutation Database (http://www.hgmd.cf.ac.uk/ac/index.php) and ClinVar database (Patel et al., 2012; Quintana et al., 2009; Sperl et al., 2015).

*PDHA1* is located in the Xp22.2‐p22.1 region, has a full length of about 17.5 kb, and contains 12 exons. Various types of mutations have been found throughout the gene sequence. Among these, missense mutations account for approximately 50% of all mutations, insertion, and deletion mutations for approximately 38% and splicing mutations for approximately 8% (Imbard et al., 2011). There are only a few reports of children with *PDHA1* mutations in China, and the genotype characteristics are still unclear. In 2007, the first case of a Chinese patient with a *PDHA1* mutation was reported with the known mutation c.214 C>T (Zhang, Sun, et al., 2007). In our case, the diagnosis was confirmed after CES, which revealed a NM_000284.4:c.1167_1170del mutation in exon 11 of *PDHA1*, resulting in the deletion of the base AGTC. The mutation in turn causes an amino acid frameshift variant, which is p.Ser390LysfsTer33. After predicting the primary, secondary, and tertiary structures of the protein by the prediction software, it was found that the protein structure has changed, and the protein terminal structure has changed. The mutation site of *PDHA1* gene had a very little incidence in the normal population database. The data in the population database were all male heterozygotes. There are some differences in the effect of *PDHA1* on the pathogenicity of different sexes. Male patients have only one X chromosome so that all cells will be affected by the *PDHA1* mutation, and the residual activity of the PDHc will depend on the severity of the mutation. There is a mutation in one of the two X chromosomes in female patients. The random X chromosome inactivation will lead to the selective expression of mutant genes and normal genes in different tissues. Therefore, the clinical manifestations of women are mainly determined by the lack of PDHc activity and the random X chromosome inactivation pattern (Deeb et al., 2014; Horga et al., 2019; Willemsen et al., 2006).

Through comprehensive analysis of database data analysis and software predictive analysis, and based on ACMG's pathogenicity assessment, we analyzed that the mutation was Pathogenic (Abou Tayoun et al., 2018; Richards et al., 2015). The specific functional changes are still unclear, and further study is needed. This mutation was not detected in the boy's parents and seemed to represent a de novo variant. Dr. Hitoshi Endo had already reported a child with this mutation in 1989. (Endo et al., 1989). Their child also had obvious clinical manifestations. Their patient mainly showed an increase in the ratio of lactate/pyruvate and was prone to exercise and fatigue at 3 years old. He got better after receiving a lot of thiamine treatment. The physical and mental development of the child was within normal limits. However, our patient's performance was more severe. He experienced recurring muscle weakness and even further developed into severe muscle weakness and stopped breathing and circulation. We used CES to prompt us to diagnose the disease better. And we used ACMG's assessment criteria to analyze the pathogenicity of the mutation in detail and determined that the mutation was pathogenic.

LS was first clinically defined in 1996, and the diagnostic criteria were amended in 2015. They now require neurological pathological changes or imaging changes, and symptoms must be accompanied by progressive neurodegenerative lesions. In addition, the criteria include (a) brainstem and/or basal ganglia dysfunction; (b) mental and motor retardation; (c) abnormal energy metabolism indicated by a severe deficiency of OXPHOS or PDHc activity, related to mitochondrial energy production; and (4) molecular gene diagnosis. If patients do not meet these criteria, such as those with atypical neurological lesions or those with normal lactate levels, a diagnosis of Leigh‐like syndrome may be considered (Rahman et al., 1996). The case presented here showed recurrent muscle weakness, progressive muscle weakness, and high blood lactate. Here, because of the limited conditions, we did not check the enzyme activity. Therefore, in addition to MRI results and enzyme activity, the patient's performance meets the diagnostic criteria. We considered that LS and Leigh‐like syndrome could be regarded as one disease continuum caused by (partially) identical pathophysiologic mechanisms, as Gerard et al. said (Gerard et al., 2016). The head MRI showed unilateral basal ganglia and midbrain lesions as well as the brainstem and cervical spinal cord lesions, and ECG showed demyelinating lesions. Signs and symptoms were thus generally in line with the clinical diagnosis of LS, and CES confirmed the diagnosis.

There is no definite effective targeted LS treatment method, but some cases have been treated (Shelkowitz et al., 2020). There are many mechanisms of LS, but it seems most important to address the specific biochemical defect (PDCD). The treatment is mainly through the following ways: (a) avoiding the energy produced by carbohydrates through KD, (b) regulating PDHc, and (c) supplementing cofactors (Sperl et al., 2015). Usually, the KD can provide ketone energy for the brain. Restricting carbohydrates can reduce the production of pyruvate, thereby reducing the production of lactic acid. The KD has also been confirmed by a large number of experiments to have a good effect on PDCD (Sofou et al., 2017; Vidali et al., 2015; Wexler et al., 1997). And for other diseases, such as epilepsy and some other mitochondrial diseases, it has a good therapeutic effect (Bedoyan et al., 2017; Branco et al., 2016; Paoli et al., 2014; Ułamek‐Kozioł et al., 2019). In addition to the classic KD treatment methods, in some studies, it has been found that the KD of medium‐chain fatty acids has a good effect in the treatment of epilepsy. However, there is no enough evidence that it is effective in LS, which needs further confirmation (Wang & Lin, 2013). In the research so far, researchers have discovered that some supplements, vitamin B1, Riboflavin (vitamin B2), and alpha‐lipoic acid, may improve mitochondrial function and neurological symptoms, but a large number of clinical trials are still needed to confirm these conclusions (Baertling et al., 2014; Gerard et al., 2016; Jauhari et al., 2017; Maesaka et al., 1976; Scarpelli et al., 2014; Sperl et al., 2015). In addition to the above therapeutic measures for PDCD, there are other nonspecific therapeutic agents such as Coenzyme Q10, Idebenone, and L‐Carnitine (Carillo et al., 2020; Chen et al., 2015; Haginoya et al., 2009; Mermigkis et al., 2013; Scarpelli et al., 2014; Wang et al., 2008). They are used for mitochondrial diseases (such as LS), but their effects may not directly affect the metabolic disorders caused by PDCD. Their more mechanisms of action are antioxidants, which promote lipid metabolism and some other effects (Finsterer, 2020).

However, not every patient can receive these treatments, and the choice should be made according to the actual situation. If patients have complications, they should be treated for comorbidities (Gerards et al., 2016). We chose the KD as the main treatment and supplemented coenzyme Q10, vitamin B1, and L‐carnitine. After more than 1 month of treatment and observation, the boy's muscle weakness had improved to a certain extent, and he could lift his upper limbs. However, 6 months later, he was still bedridden and unable to stand.

## 4 CONCLUSION

To summarize, our observations in this 12‐year‐old boy suggest that the gene mutation is pathogenic, and the popularization of gene detection may help improve the diagnosis accuracy of LS. Although there is currently no effective treatment, the continuation of relevant research and the emergence of new technologies, genetic diagnostic tools, and gene therapy approaches may also provide a cure for LS in the future.

## CONFLICT OF INTEREST

The authors declare that they have no competing interests.

## AUTHOR CONTRIBUTIONS

KG collected the data and wrote the manuscript. LX, ZSW, XX, and XXZ conducted the treatment. JLC conducted the treatment, the data analysis, and made all necessary modifications to the manuscript. The final draft was read and approved by all authors.

## Data Availability

The datasets used and/or analyzed during the current study are available from the corresponding author on reasonable request.
